# Associations between Food Preferences, Food Approach, and Food Avoidance in a Polish Adolescents’ COVID-19 Experience (PLACE-19) Study Population

**DOI:** 10.3390/nu13072427

**Published:** 2021-07-15

**Authors:** Dominika Guzek, Dominika Skolmowska, Dominika Głąbska

**Affiliations:** 1 Department of Food Market and Consumer Research, Institute of Human Nutrition Sciences, Warsaw University of Life Sciences (SGGW-WULS), 159C Nowoursynowska Street, 02-776 Warsaw, Poland; 2 Department of Dietetics, Institute of Human Nutrition Sciences, Warsaw University of Life Sciences (SGGW-WULS), 159C Nowoursynowska Street, 02-776 Warsaw, Poland; dominika_skolmowska@sggw.edu.pl (D.S.); dominika_glabska@sggw.edu.pl (D.G.)

**Keywords:** food preferences, food approach, food avoidance, appetitive traits, Food Preference Questionnaire (FPQ), Adult Eating Behaviour Questionnaire (AEBQ), adolescents, national study, population-based study, PLACE-19 Study

## Abstract

Food preferences are among the strongest predictors of the food choices of adolescents. These are associated with appetitive traits (food approach and avoidance) to some extent. However, no research has been conducted so far analyzing the association between food preferences and appetitive traits of adolescents. The aim of this study was to evaluate the associations between food preferences and appetitive traits in adolescents (aged 15–20 years) within the Polish Adolescents’ COVID-19 Experience (PLACE-19) Study population. The PLACE-19 Study was carried out in a population-based sample of 2448 secondary school students sampled across the country (random quota sampling). Food preferences (including the preference for vegetables, fruit, meat/fish, dairy, snacks, and starches) of the adolescents were assessed using the validated Food Preference Questionnaire (FPQ) while their appetitive traits (hunger, food responsiveness, emotional overeating, enjoyment of food, satiety responsiveness, emotional undereating, food fussiness, slowness in eating) were assessed using the validated Adult Eating Behavior Questionnaire (AEBQ). The k-means clustering was performed to identify the homogenous clusters of respondents based on their preferences, and linear regression was performed to determine the relationship between food preferences and appetitive traits with a model adjusted for sex and age. Based on their preferences, three homogenous clusters of respondents were defined: low-preferring respondents (low preference for all food categories), respondents preferring snacking foods (low preference for all food categories, except for fruit and snacks), and high-preferring respondents (high preference for all food categories). The low-preferring respondents showed the lowest values for all appetitive traits (*p* = 0.0008), as well as the lowest total score (*p* = 0.0001), except for food fussiness, for which they showed the highest value (*p* = 0.0008). All preference scores were positively associated with traits such as hunger, food responsiveness, enjoyment of food, and emotional under-eating, while negatively associated with food fussiness (all *p* < 0.05). The largest amount of variance was observed for preference for dairy (14.6%; *R*^2^ = 0.146, *p* = 0.008) and snacks with respect to enjoyment of food (16.2%; *R*^2^ = 0.162, *p* = 0.008), for vegetable with respect to food fussiness (22%; *R*^2^ = 0.220, *p* = 0.008), and for meat/fish with respect to enjoyment of food (19.9%; *R*^2^ = 0.199, *p* = 0.008) and food fussiness combined (19.1%; *R*^2^ = 0.191, *p* = 0.008). These results support the association of food preferences with both food approach traits and food avoidance traits.

## 1. Introduction

As indicated by the World Health Organization (WHO), adolescence is a transitional period between childhood and adulthood, which allows preventing nutrition-related chronic diseases in the future, while addressing specific nutritional issues and correcting those that originated in the past [1]. However, in this period, individuals prioritize personal preferences over family eating habits, as they have progressively more control over their own diet [2]. It has been stated that self-reported food preferences are among the strongest predictors of the food choices of adolescents [3]. Thus, understanding food preferences is necessary to promote healthy eating patterns in this population [4]. Several studies conducted among youths have confirmed the role of food preferences as a powerful determinant of diet and consequently health. Their results indicate that food preferences influence weight status [5] and nutritional risk factors of diet-related diseases [6].

Food preferences, associated with the acceptability of food products, are defined as evaluative attitudes expressed by people toward foods including how they qualitatively evaluate them and how much they like or dislike specific products [7]. These preferences are established from early childhood [8]. The development of food preferences is influenced by multiple factors, including personal ones (e.g., innate preferences, applied feeding practices), as well as those associated with parents (e.g., maternal diet during pregnancy and breastfeeding), community (e.g., restaurants, food retailers), and macroenvironment (e.g., food marketing, media) [6]. The possibility of modifying the developed food preferences is limited, as they remain stable until adulthood [9]. However, during the lifetime of an individual, acuity and discriminating ability of senses change, which also lead to some changes in taste and smell perceptions and as a result modify food preferences [10].

The other key determinants of food choices are appetitive traits [11], which are also associated with food preferences to some extent. Appetitive traits are defined as persistent predispositions toward food [12]. They include food approach traits (hunger, food responsiveness, emotional over-eating, enjoyment of food) and food avoidance traits (satiety responsiveness, emotional under-eating, food fussiness, slowness in eating) [13]. A study conducted on children aged 3–4 years indicated that preference for fruit or vegetables is positively associated with enjoyment of food and negatively associated with satiety responsiveness, slowness in eating, and food fussiness [14]. Similarly, another study conducted on children aged 2–5 years showed that preference for fruit or vegetables is positively associated with enjoyment of food and negatively associated with food fussiness [15]. However, it should be mentioned that in both studies [14,15], parents reported the preferences of their children and the preferences were not self-reported.

As previous studies analyzed only children, but not adolescents, the associations in older age groups are still unknown. Moreover, even in the case of children, the authors of the studies emphasize that further research should be performed to describe the causal mechanisms driving the associations between appetitive traits and food preferences [14], as well as to evaluate when, how, and why some appetitive traits are associated with food preferences [15].

Taking into account the COVID-19 pandemic, it must be noted that the present global situation may change food priorities [16]. Appetitive traits also appear to be influenced by the pandemic, as it was reported that in this period students experience moderate-to-severe anxiety symptoms due to increased hunger, emotional over-eating, and food and satiety responsiveness, as well as decreased enjoyment of food [17]. Thus, the associations of food preferences and appetitive traits may also be modified, and so they should be studied in the period of the COVID-19 pandemic. Moreover, as it is predicted that 1.58–8.76 million COVID-19 deaths can be observed over 5 years until the end of 2024 [18], we must be prepared for the prolonged pandemic and more studies should be conducted during this period [19].

Considering the above, the aim of the present study was to verify the association between food preferences and appetitive traits in adolescents aged 15–20 years within the Polish Adolescents’ COVID-19 Experience (PLACE-19) Study population.

## 2. Materials and Methods

### 2.1. Ethical Statement

The PLACE-19 Study was carried out at the Institute of Human Nutrition Sciences, Warsaw University of Life Sciences (WULS-SGGW). It was conducted based on the agreement of the Ethics Committee of the Institute of Human Nutrition Sciences of the Warsaw University of Life Sciences. The participants, as well as their parents/legal guardians, provided informed consent for participation, and all the procedures were in agreement with the Declaration of Helsinki. The PLACE-19 Study included two phases to assess hygienic and personal protective behaviors [20,21,22], as well as nutritional behaviors [23,24,25].

### 2.2. Studied Population

To assess nutritional behaviors within the second phase of the PLACE-19 Study, the studied population was recruited in the period from 29 April 2020 to 23 May 2020. The studied group was population-based and it was sampled in whole Poland, while using a random quota sampling method. As described in the previous studies [23,24,25], the procedure included stratified random sampling of counties within voivodeships (being the Polish basic administrative units), followed by random sampling of schools within counties, in order to gather population-based sample including proportional share of adolescents from all regions. While arranging, the local Boards of Education participated, if needed, but the participation of school was for its headmaster voluntary, as well as individual participation was for each student voluntary.

The inclusion criteria were formulated as follows:

− Students of the sampled school,
− Aged 15–20 years,
− Providing informed consent to participate in the PLACE-19 Study, as well as informed consent of parents/legal guardians.
− The exclusion criteria were formulated as follows:
− Any missing or unreliable data in the questionnaire,
− For the participants of the second phase of the PLACE-19 Study (assessment of nutritional behaviors): not participating in the first phase of the PLACE-19 Study (assessment of hygienic and personal protective behaviors).

The second phase of the PLACE-19 Study was finally carried out in a sample of 2448 secondary school students which were included based on the presented inclusion and exclusion criteria, as described in the previous studies [23,24,25].

### 2.3. Applied Questionnaires

As in the period from April to May 2020, on the basis of the decision of Polish Ministry of Education [26] education in all secondary schools in Poland was provided within a remote learning system, the study was conducted while using a method of Computer-Assisted Web Interview (CAWI). The questionnaire did not collect any sensitive or personal data which would allow to recognize a respondent, either for researchers, or for school headmasters and teachers.

The food preferences were assessed while using Food Preference Questionnaire (FPQ) by Smith et al. [27]. The FPQ is a validated self-report tool to be applied in case of children and adolescents which was developed by Smith et al. [27] based on the previously validated tool for children to be proxy-reported by parents [28]. The FPQ includes a list of 62 various food items to be defined on how much on average the respondent like the specific item with the possible answers as follows: (1) dislike a lot, (2) dislike a little, (3) neither like nor dislike, (4) like a little, (5) like a lot (for any food item they have ever tried, independently from the actual consumption), as well as (6) not applicable (for any food item they don’t know, or don’t remember ever having tried) [29]. The FPQ allows to assess the preferences of vegetables (questionnaire includes 18 food items in this food category), fruit (7 items), meat/fish (12 items), dairy (10 items), snacks (9 items), and starches (6 items), which may be obtained by summing the single food preference item scores within each food category and dividing this sum by the number of items [29].

The appetitive traits were assessed while using Adult Eating Behavior Questionnaire (AEBQ) by Hunot et al. [13]. The AEBQ is a validated self-report tool to be applied in case of adolescents and adults which was developed to assess food approach and food avoidance. The AEBQ includes a list of 35 items to be defined while using a 5-point Likert scale (from ‘strongly disagree’ to ‘strongly agree’). The AEBQ allows to assess following food approach sub-scales: hunger (5 items), food responsiveness (4 items), emotional over-eating (5 items), enjoyment of food (3 items) and following food avoidance sub-scales: satiety responsiveness (4 items), emotional under-eating (5 items), food fussiness (5 items), slowness in eating (4 items). The scores for each scale are obtained by attributing points to each item–depending on a question either from 1 to 5 points, or from 5 to 1 point, and by calculating mean score for each subscale.

### 2.4. Statistical Analysis

The normality of distribution was verified by using Shapiro-Wilk test. Due to nonparametric distribution, the Kruskal–Wallis analysis of variance (ANOVA) with post-hoc Tukey test was applied to compare groups and Spearman correlation for analysis of associations within heatmap of correlation matrix. Based on the assumption that the individual food product preferences do not present equally in the entire population and that specific consumer groups typically differ within the population, presenting various profiles, the clustering of respondents based on their preferences was conducted. For clustering, k-means algorithm was applied with Euclidean distance, as generally recommended [30], to identify homogenous clusters of respondents based on their preferences, while the optimal number of clusters was verified using the Elbow method. For analysis of associations, the linear regression between food preferences and appetitive traits was performed with model adjusted for sex and age, as this approach was confirmed by the previous studies as adequate [14]. The standardized β-coefficients were presented in order to allow the comparison of results between the scores. As an additional analysis, the multiple linear regression was applied and F-statistics was presented for the variables indicated as significant based on the previous analysis. Due to the multiple analysis conducted, Bonferroni correction was applied.

The statistical significance was defined for the level of *p* ≤ 0.05. The statistical analysis was performed while using Statistica version 13.3 (StatSoft Inc., Tulsa, OK, USA) and JASP version 0.14.0.0 (JASP Team, 2020).

## 3. Results

The characteristics of the population studied within the second phase of the PLACE-19 Study is presented in Table 1. The sex and age were indicated as the most important variables and in the further analysis they were included to the model.

The food preferences assessed by FPQ within the population of the second phase of the PLACE-19 Study are presented in Table 2. The highest preferences in the studied group were defined for the categories of fruit, snacks and starches (median values higher than 4 points attributed to ‘like a little’ category) and the lowest—for the category of meat/fish (median value higher than 3 points attributed to ‘neither like nor dislike’ category).

**Figure 1:**
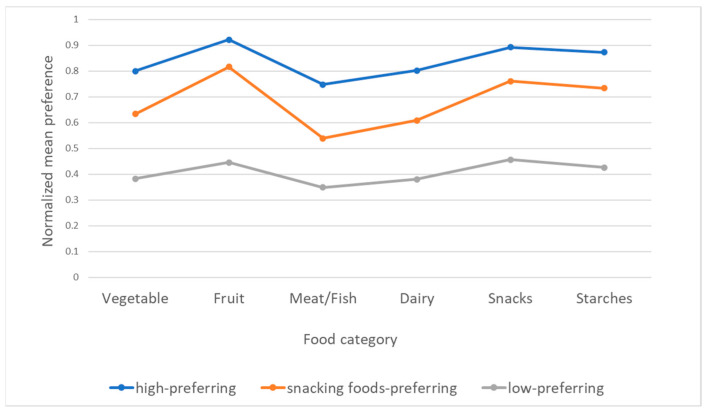
Normalized mean values for the preference within the sub-groups stratified based on the clustering of the preferences assessed by Food Preference Questionnaire (FPQ) within the population of the second phase of the Polish Adolescents’ COVID-19 Experience (PLACE-19) Study (*n* = 2419).

The food preferences for sub-groups stratified based on the clustering of the preferences assessed by FPQ are presented in Table 3 and the normalized mean values for the preferences within the sub-groups stratified based on the clustering of the preferences assessed by FPQ within the population of the second phase of the PLACE-19 Study are presented in Figure 1. While the k-means algorithm was applied, 3 homogenous clusters of respondents were defined, based on their preferences—low-preferring respondents (low preference for all food categories), respondents preferring snacking foods (low preference for all food categories, except for fruit and snacks, as for them median values were higher than 4 points attributed to ‘like a little’ category), and high-preferring respondents (high preference for all food categories, as median values were higher than 4 points attributed to ‘like a little’ category).

The appetitive traits assessed by AEBQ for sub-groups stratified based on the clustering of the preferences assessed by FPQ within the population of the second phase of the PLACE-19 Study are presented in Table 4. All appetitive traits differed between respondents from various clusters. The low-preferring respondents (cluster 1) were characterized by the lowest values for all appetitive traits (*p* = 0.0008), as well as for the total score (*p* = 0.0001), except for food fussiness, as for it this cluster was characterized by the highest value (*p* = 0.0008). The snacking-preferring respondents (cluster 2) and high-preferring ones (cluster 3) were characterized by comparable traits of hunger and emotional overeating, while all other appetitive traits differed significantly (*p* = 0.0008).

Analysis of association between appetitive traits and vegetable preference assessed by FPQ, in model adjusted for sex and age, within the population of the second phase of the PLACE-19 Study is presented in Table 5. Vegetable preference score was positively associated with hunger, food responsiveness, emotional over-eating, enjoyment of food, emotional under-eating, and slowness in eating, while negatively associated with food fussiness. Food fussiness explained the largest amount of variance (22%, R^2^ = 0.220, *p* = 0.008) for the studied population. The appetitive traits that were significantly associated with the vegetable preference were included to the regression model with sex and age as additional demographic variables (R^2^ = 0.26, F(82,406) = 106.91, *p* = 0.001).

Analysis of association between appetitive traits and fruit preference assessed by FPQ, in model adjusted for sex and age, within the population of the second phase of the PLACE-19 Study is presented in Table 6. Fruit preference score was positively associated with hunger, food responsiveness, enjoyment of food, satiety responsiveness, and emotional under-eating, while negatively associated with food fussiness. Enjoyment of food and food fussiness explained the largest amount of variance (7.6%, R^2^ = 0.076, *p* = 0.008 and 7.7%, R^2^ = 0.077, *p* = 0.008, respectively) for the studied population. The appetitive traits that were significantly associated with the fruit preference were included to the regression model with sex and age as additional demographic variables (R^2^ = 0.14, F(82,321) = 46.85, *p* = 0.001).

Analysis of association between appetitive traits and meat/fish preference assessed by FPQ, in model adjusted for sex and age, within the population of the second phase of the PLACE-19 Study is presented in Table 7. Meat/fish preference score was positively associated with hunger, food responsiveness, enjoyment of food, and emotional under-eating, while negatively associated with food fussiness. Enjoyment of food and food fussiness explained the largest amount of variance (19.9%, R^2^ = 0.199, *p* = 0.008 and 19.1%, R^2^ = 0.191, *p* = 0.008, respectively) for the studied population. The appetitive traits that were significantly associated with the meat/fish preference were included to the regression model with sex and age as additional demographic variables (R^2^ = 0.27, F(62,418) = 145.62, *p* = 0.001).

Analysis of association between appetitive traits and dairy preference assessed by FPQ, in model adjusted for sex and age, within the population of the second phase of the PLACE-19 Study is presented in Table 8. Dairy preference score was positively associated with hunger, food responsiveness, emotional over-eating, enjoyment of food, and emotional under-eating, while negatively associated with food fussiness. Enjoyment of food explained the largest amount of variance (14.6%, R^2^ = 0.146, *p* = 0.008) for the studied population. The appetitive traits that were significantly associated with the dairy preference were included to the regression model with sex and age as additional demographic variables (R^2^ = 0.17, F(82,408) = 64.33, *p* = 0.001).

Analysis of association between appetitive traits and snacks preference assessed by FPQ, in model adjusted for sex and age, within the population of the second phase of the PLACE-19 Study is presented in Table 9. Snacks preference score was positively associated with hunger, food responsiveness, emotional over-eating, enjoyment of food, satiety responsiveness, and emotional under-eating, while negatively associated with food fussiness. Enjoyment of food explained the largest amount of variance (16.2%, R^2^ = 0.162, *p* = 0.008) for the studied population. The appetitive traits that were significantly associated with the snacks preference were included to the regression model with sex and age as additional demographic variables (R^2^ = 0.18, F(82,332) = 66.23, *p* = 0.001).

Analysis of association between appetitive traits and starches preference assessed by FPQ, in model adjusted for sex and age, within the population of the second phase of the PLACE-19 Study is presented in Table 10. Starches preference score was positively associated with hunger, food responsiveness, emotional over-eating, enjoyment of food, satiety responsiveness, and emotional under-eating, while negatively associated with food fussiness. Enjoyment of food explained the largest amount of variance (8.9%, R^2^ = 0.089, *p* = 0.008) for the studied population. The appetitive traits that were significantly associated with the starches preference were included to the regression model with sex and age as additional demographic variables (R^2^ = 0.12, F(92,358) = 36.36, *p* = 0.001).

**Figure 2:**
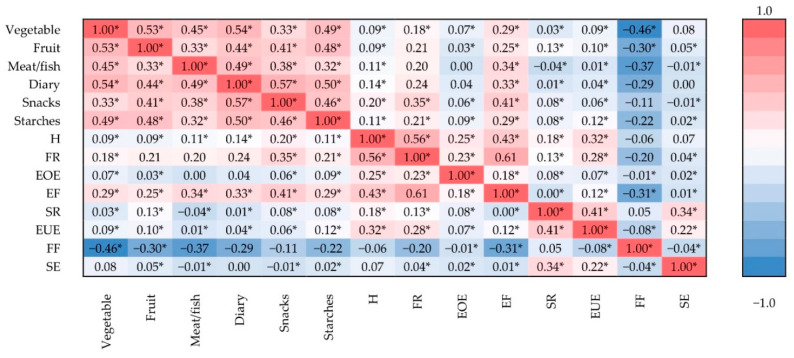
Heatmap of correlation matrix presenting association between food preferences assessed by Food Preference Questionnaire (FPQ) and appetitive traits assessed by Adult Eating Behavior Questionnaire (AEBQ), conducted while using Spearman correlation (due to nonparametric distribution), within the population of the second phase of the Polish Adolescents’ COVID-19 Experience (PLACE-19) Study (*n* = 2419); H—Hunger; FR—Food responsiveness; EOE—Emotional over-eating; EF—Enjoyment of food; SR—Satiety responsiveness; EUE—Emotional under-eating; FF—Food fussiness; SE—Slowness in eating; * significant association (*p* ≤ 0.05).

Heatmap of correlation matrix presenting association between food preferences assessed by FPQ and appetitive traits assessed by AEBQ, within the population of the second phase of the PLACE-19 Study is presented in Figure 2. This unadjusted analysis confirmed the weakest associations or negative associations for food fussiness, while for other appetitive traits the associations were more significant.

## 4. Discussion

The study assessed the preference for vegetables, fruit, meat/fish, dairy, snacks, and starches in a group of Polish adolescents. The preference scores for all food categories were found to be positively associated with hunger, food responsiveness, enjoyment of food, and emotional under-eating, as well as negatively associated with food fussiness. The associations between appetitive traits and general food preferences may suggest that these features are predetermined. As indicated by Fildes et al. [14], it appears that appetitive traits associated with an increased risk of excessive body mass may have additional lifelong consequences and also influence dietary diversity and nutrient intake.

As mentioned above, hunger, food responsiveness, and enjoyment of food are food approach traits, while emotional under-eating and food fussiness are food avoidance traits [31]. Taking this into account, it may be indicated that the association between food preferences and appetitive traits is not influenced by the type of appetitive traits (approach or avoidance), but other factors may more likely determine the associations between food preferences and food fussiness compared to other appetitive traits.

Food fussiness is an appetitive trait described as refusing and not tasting new foods at first, disliking a food before tasting it, not enjoying a wide variety of foods, as well as not being interested in tasting new foods that have not been tried before [13]. This trait is observed to differ from the other food avoidance as well as other appetitive traits, as in adults it is not associated with Body Mass Index (BMI), although in general BMI is positively associated with food approach traits [32] and negatively associated with food avoidance traits [13,32]. The difference can be explained by the fact that food fussiness is qualitatively distinct as it reflects a food choice selectivity, while the other food avoidance traits reflect decreased appetite and increased sensitivity to satiety cues [33].

Previous studies conducted on children have also confirmed that food fussiness was negatively associated with food preferences. In children aged 2–5 years, it was negatively associated with preference for vegetables, fruit, extra foods (those not essential to provide the nutrients needed by the body, and hence similar to snacks as indicated in the present study), dairy, meats, cereals, and a number of liked foods, while it was positively associated with a number of disliked foods [15]. At the same time, in children aged 3–4 years, this trait was negatively associated with the preference for vegetables and fruit, but not associated with the preference for noncore foods (energy-dense and nutrient-poor discretionary foods, and hence similar to snacks as indicated in the present study) [14]. The lack of association between food fussiness and snacks in the case of the youngest children may be, to some extent, related to the fact that this trait seems to decrease with age [34], and hence, its association with food preference may also change during the lifespan.

The positive associations observed between food preferences and hunger, food responsiveness, enjoyment of food, as well as emotional under-eating in this study are in line with the results of the previous studies by other authors. In the study on children aged 2–5 years, emotional under-eating was positively associated with the preference for vegetables, extra foods, dairy, meats, and cereals; enjoyment of food was positively associated with the preference for vegetables, fruit, dairy, meats, and cereals, but negatively associated with the preference for extra foods; and food responsiveness was positively associated with preference for extra foods, but negatively with the preference for vegetables [15]. On the other hand, in the study on children aged 3–4 years, food responsiveness was positively associated with the preference for vegetables and fruit, while enjoyment of food was positively associated with the preference for noncore foods [16].

Considering the role of appetitive traits, it seems that they may be crucial for choosing and consuming specific food products. Thus, based on the present study, it can be assumed that adolescents with higher food fussiness may reject some products, especially fruit and vegetables [35]. Similarly, adolescents with higher food responsiveness and enjoyment of food may eagerly try novel, previously unknown food products, which may lead to an increased preference and intake of fruit and vegetables [36].

Similarly, the analysis of the clusters of low-preferring respondents, respondents preferring snacking foods, and high-preferring respondents indicated the influence of appetitive traits on low-preferring respondents, as they had the lowest values for all appetitive traits, except for food fussiness, for which they showed the highest value. The number of respondents in this cluster was low (only 11%), and they may be generally defined as picky eaters, who reject or restrict both familiar and unfamiliar foods [37], while their food fussiness was significantly higher compared to the other clusters. At the same time, the other clusters showed a high preference for fruit and snacks only (respondents preferring snacking foods) or a high preference for all products in general. Taking this into account, the preference and resultant intake of fruit and vegetables may be supposed to be higher only in the high-preferring cluster, which may be considered a public health problem due to the potential benefits of fruit and vegetables [38], as well as the inadequate intake of fruit and vegetables in the case of adolescents [39].

It should be indicated that the present study assessed only the preferences and not the intake of fruit and vegetables. However, the findings of a recent systematic review and meta-analysis by Bawajeeh et al. [40], which analyzed the association between taste, preference, and food choices in adolescents, emphasized that food choices and intake are created by a complex combination of various factors, which should be studied in detail to expand the existing knowledge.

The present study not only reproduced, for a Polish cohort, specific associations between appetitive traits and food preferences defined by previous studies [14,15], but also confirmed the in-depth observations reported by their authors. Such observations are based on positive associations between food approach traits and food preferences. As food approach traits have been assumed to promote excessive body mass, their association with a higher preference for various products may be an additional factor promoting overconsumption. This is an important issue to be taken into account, as it may have lifelong consequences on the quality of diet and body mass.

## 5. Conclusions

This study showed that all preference scores were associated with both food approach and food avoidance traits. They were positively associated with hunger, food responsiveness, enjoyment of food, and emotional under-eating, while negatively associated with food fussiness. The largest amount of variance was observed for the preference for dairy and snacks with respect to enjoyment of food, for vegetables with respect to food fussiness, and for meat/fish with respect to enjoyment of food and food fussiness combined.

## Figures and Tables

**Table 1:** The characteristics of the population studied within the second phase of the Polish Adolescents’ COVID-19 Experience (PLACE-19) Study (*n* = 2448).

Variable		Number of Participants	% of the Studied Population
Sex	Female	1552	63.4
	Male	896	36.6
Age (years)	15	297	12.1
	16	755	30.8
	17	799	32.6
	18	445	18.2
	19	139	5.7
	20	13	0.5

**Table 2:** The food preferences assessed by Food Preference Questionnaire (FPQ) within the population of the second phase of the Polish Adolescents’ COVID-19 Experience (PLACE-19) Study (*n* = 2448) (distribution different than normal for all the variables verified using Shapiro-Wilk test—*p* ≤ 0.05).

Food Category	Mean ± SD	Median	25th Quartile	75th Quartile
Vegetable	3.7 ± 0.7	3.8	3.2	4.3
Fruit	4.3 ± 0.8	4.6	4.0	5.0
Meat/fish	3.4 ± 0.8	3.5	2.9	4.0
Dairy	3.7 ± 0.7	3.8	3.2	4.2
Snacks	4.2 ± 0.8	4.3	3.8	4.8
Starches	4.0 ± 0.8	4.2	3.7	4.7

**Table 3:** The food preferences for sub-groups stratified based on the clustering of the preferences assessed by Food Preference Questionnaire (FPQ) within the population of the second phase of the Polish Adolescents’ COVID-19 Experience (PLACE-19) Study (*n* = 2419) (distribution different than normal for all the variables, except for dairy for cluster 1, verified using Shapiro-Wilk test—*p* ≤ 0.05).

Food Category	Cluster 1 (Low-Preferring) (*n* = 270)		Cluster 2 (Snacking Foods-Preferring) (*n* = 1109)		Cluster 3 (High-Preferring) (*n* = 1040)	
Vegetable	2.5 ± 0.6	2.5 (2.1–2.9)	3.5 ± 0.6	3.6 (3.2–3.9)	4.2 ± 0.5	4.2 (3.9–4.6)
Fruit	2.8 ± 0.8	3.0 (2.1–3.0)	4.3 ± 0.6	4.3 (4.0–4.8)	4.7 ± 0.4	4.9 (4.6–5.0)
Meat/fish	2.4 ± 0.7	2.3 (1.9–2.9)	3.2 ± 0.7	3.3 (2.8–3.6)	4.0 ± 0.6	4.0 (3.6–4.4)
Dairy	2.5 ± 0.6	2.6 (2.1–2.9)	3.4 ± 0.5	3.4 (3.1–3.8)	4.2 ± 0.5	4.2 (3.9–4.6)
Snacks	2.8 ± 0.7	2.9 (2.4–3.1)	4.1 ± 0.6	4.1 (3.8–4.6)	4.6 ± 0.5	4.8 (4.3–5.0)
Starches	2.7 ± 0.7	2.8 (2.2–3.0)	3.9 ± 0.6	4.0 (3.7–4.3)	4.5 ± 0.5	4.5 (4.2–4.8)

**Table 4:** The appetitive traits assessed by Adult Eating Behavior Questionnaire (AEBQ) for sub-groups stratified based on the clustering of the preferences assessed by Food Preference Questionnaire (FPQ) within the population of the second phase of the Polish Adolescents’ COVID-19 Experience (PLACE-19) Study (*n* = 2419) (distribution different than normal for all the variables verified using Shapiro-Wilk test—*p* ≤ 0.05).

Appetitive Traits	Cluster 1 (Low-Preferring) (*n* = 270)		Cluster 2 (Snacking Foods-Preferring) (*n* = 1109)		Cluster 3 (High-Preferring) (*n* = 1040)		*p*
Food approach subscales							
Hunger (H)	2.3 ± 0.5	2.0 (2.0–2.4) ^a^	2.5 ± 0.6	2.4 (2.0–2.8) ^b^	2.5 ± 0.6	2.4 (2.0–2.8) ^b^	0.0008
Food responsiveness (FR)	2.3 ± 0.6	2.0 (2.0–2.5) ^a^	2.8 ± 0.6	2.8 (2.5–3.3) ^b^	3.0 ± 0.7	3.0 (2.5–3.3) ^c^	0.0008
Emotional over-eating (EOE)	2.6 ± 0.3	2.6 (2.4–2.6) ^a^	2.8 ± 0.6	2.6 (2.4–3.0) ^b^	2.8 ± 0.6	2.6 (2.4–3.0) ^b^	0.0008
Enjoyment of food (EF)	2.5 ± 0.8	2.0 (2.0–3.0) ^a^	3.6 ± 0.8	3.7 (3.0–4.0) ^b^	3.9 ± 0.8	4.0 (3.3–4.3) ^c^	0.0008
Food avoidance subscales							
Satiety responsiveness (SR)	2.5 ± 0.8	2.0 (2.0–3.0) ^a^	3.0 ± 0.7	3.0 (2.5–3.5) ^b^	2.8 ± 0.7	2.8 (2.3–3.3) ^c^	0.0008
Emotional under-eating (EUE)	2.5 ± 0.8	2.0 (2.0–2.8) ^a^	2.9 ± 0.8	2.6 (2.0–3.4) ^b^	2.8 ± 0.8	2.6 (2.0–3.2) ^c^	0.0008
Food fussiness (FF)	3.2 ± 0.5	3.2 (3.2–3.2) ^a^	2.8 ± 0.8	2.8 (2.2–3.4) ^b^	2.4 ± 0.7	2.2 (1.8–2.8) ^c^	0.0008
Slowness in eating (SE)	2.8 ± 0.6	2.5 (2.5–3.0) ^a^	3.0 ± 0.8	3.0 (2.5–3.5) ^b^	2.9 ± 0.8	2.8 (2.3–3.5) ^c^	0.0008
Total	2.6 ± 0.4	2.3 (2.3–2.9) ^a^	2.9 ± 0.3	2.9 (2.7–3.1) ^b^	2.8 ± 0.3	2.8 (2.6–3.0) ^c^	0.0001

^a,b,c^—values marked with different letters in rows differ significantly.

**Table 5:** Analysis of association between appetitive traits and vegetable preference assessed by Food Preference Questionnaire (FPQ) (model adjusted for sex and age) within the population of the second phase of the Polish Adolescents’ COVID-19 Experience (PLACE-19) Study (*n* = 2415).

Appetitive Traits	Unstandardized Coefficients		Standardized Coefficients β	*p*	R	R^2^
	β	SE				
Hunger (H)	0.108	0.025	0.087	0.008	0.132	0.017
Food responsiveness (FR)	0.201	0.022	0.185	0.008	0.210	0.044
Emotional over-eating (EOE)	0.118	0.026	0.092	0.008	0.135	0.018
Enjoyment of food (EF)	0.258	0.015	0.321	0.008	0.336	0.113
Satiety responsiveness (SR)	0.014	0.021	0.014	1.000	0.101	0.010
Emotional under-eating (EUE)	0.060	0.018	0.070	0.008	0.121	0.015
Food fussiness (FF)	−0.427	0.017	−0.459	0.008	0.469	0.220
Slowness in eating (SE)	0.058	0.019	0.062	0.024	0.117	0.014

SE—standard error.

**Table 6:** Analysis of association between appetitive traits and fruit preference assessed by Food Preference Questionnaire (FPQ) (model adjusted for sex and age) within the population of the second phase of the Polish Adolescents’ COVID-19 Experience (PLACE-19) Study (*n* = 2330).

Appetitive Traits	Unstandardized Coefficients		Standardized Coefficients β	*p*	R	R^2^
	β	SE				
Hunger (H)	0.082	0.023	0.075	0.008	0.096	0.009
Food responsiveness (FR)	0.183	0.020	0.189	0.008	0.197	0.039
Emotional over-eating (EOE)	0.031	0.024	0.027	1.000	0.065	0.004
Enjoyment of food (EF)	0.197	0.015	0.269	0.008	0.275	0.076
Satiety responsiveness (SR)	0.102	0.019	0.112	0.008	0.125	0.016
Emotional under-eating (EUE)	0.056	0.016	0.073	0.008	0.093	0.009
Food fussiness (FF)	−0.226	0.017	−0.272	0.008	0.278	0.077
Slowness in eating (SE)	0.027	0.017	0.033	0.960	0.068	0.005

SE—standard error.

**Table 7:** Analysis of association between appetitive traits and meat/fish preference assessed by Food Preference Questionnaire (FPQ) (model adjusted for sex and age) within the population of the second phase of the Polish Adolescents’ COVID-19 Experience (PLACE-19) Study (*n* = 2425).

Appetitive Traits	Unstandardized Coefficients		Standardized Coefficients β	*p*	R	R^2^
	β	SE				
Hunger (H)	0.126	0.028	0.088	0.008	0.278	0.077
Food responsiveness (FR)	0.250	0.024	0.201	0.008	0.332	0.110
Emotional over-eating (EOE)	0.038	0.029	0.026	1.000	0.265	0.070
Enjoyment of food (EF)	0.331	0.017	0.361	0.008	0.446	0.199
Satiety responsiveness (SR)	0.032	0.024	0.027	1.000	0.265	0.070
Emotional under-eating (EUE)	0.058	0.020	0.059	0.032	0.270	0.073
Food fussiness (FF)	−0.373	0.020	−0.349	0.008	0.437	0.191
Slowness in eating (SE)	0.040	0.021	0.038	0.472	0.267	0.071

SE—standard error.

**Table 8:** Analysis of association between appetitive traits and dairy preference assessed by Food Preference Questionnaire (FPQ) (model adjusted for sex and age) within the population of the second phase of the Polish Adolescents’ COVID-19 Experience (PLACE-19) Study (*n* = 2417).

Appetitive Traits	Unstandardized Coefficients		Standardized Coefficients β	*p*	R	R^2^
	β	SE				
Hunger (H)	0.190	0.025	0.152	0.008	0.209	0.044
Food responsiveness (FR)	0.278	0.021	0.254	0.008	0.291	0.085
Emotional over-eating (EOE)	0.098	0.026	0.076	0.008	0.162	0.026
Enjoyment of food (EF)	0.287	0.015	0.354	0.008	0.382	0.146
Satiety responsiveness (SR)	0.036	0.021	0.035	0.720	0.148	0.022
Emotional under-eating (EUE)	0.059	0.018	0.069	0.008	0.158	0.025
Food fussiness (FF)	−0.249	0.018	−0.264	0.008	0.300	0.090
Slowness in eating (SE)	0.031	0.019	0.033	0.832	0.147	0.022

SE—standard error.

**Table 9:** Analysis of association between appetitive traits and snacks preference assessed by Food Preference Questionnaire (FPQ) (model adjusted for sex and age) within the population of the second phase of the Polish Adolescents’ COVID-19 Experience (PLACE-19) Study (*n* = 2341).

Appetitive Traits	Unstandardized Coefficients		Standardized Coefficients β	*p*	R	R^2^
	β	SE				
Hunger (H)	0.182	0.023	0.164	0.008	0.172	0.030
Food responsiveness (FR)	0.312	0.019	0.318	0.008	0.322	0.103
Emotional over-eating (EOE)	0.084	0.024	0.074	0.008	0.092	0.009
Enjoyment of food (EF)	0.296	0.014	0.399	0.008	0.403	0.162
Satiety responsiveness (SR)	0.070	0.020	0.075	0.008	0.093	0.009
Emotional under-eating (EUE)	0.034	0.016	0.044	0.328	0.070	0.005
Food fussiness (FF)	−0.075	0.017	−0.089	0.008	0.105	0.011
Slowness in eating (SE)	0.012	0.018	0.014	1.000	0.058	0.003

SE—standard error.

**Table 10:** Analysis of association between appetitive traits and starches preference assessed by Food Preference Questionnaire (FPQ) (model adjusted for sex and age) within the population of the second phase of the Polish Adolescents’ COVID-19 Experience (PLACE-19) Study (*n* = 2368).

Appetitive Traits	Unstandardized Coefficients		Standardized Coefficients β	*p*	R	R^2^
	β	SE				
Hunger (H)	0.112	0.025	0.093	0.008	0.099	0.010
Food responsiveness (FR)	0.199	0.021	0.189	0.008	0.191	0.036
Emotional over-eating (EOE)	0.122	0.025	0.099	0.008	0.103	0.011
Enjoyment of food (EF)	0.233	0.015	0.297	0.008	0.298	0.089
Satiety responsiveness (SR)	0.076	0.021	0.077	0.008	0.082	0.007
Emotional under-eating (EUE)	0.080	0.017	0.098	0.008	0.100	0.010
Food fussiness (FF)	−0.191	0.018	−0.213	0.008	0.215	0.046
Slowness in eating (SE)	0.022	0.019	0.025	1.000	0.041	0.002

Models adjusted for covariates including sex and age; SE—standard error.
